# Clinician views and experiences of non‐invasive prenatal genetic screening tests in Australia

**DOI:** 10.1111/ajo.13533

**Published:** 2022-05-10

**Authors:** Shannon McKinn, Nasrin Javid, Ainsley J. Newson, Lucinda Freeman, Carissa Bonner, Antonia W. Shand, Natasha Nassar, Katy J.L. Bell

**Affiliations:** ^1^ Wiser Healthcare, Sydney School of Public Health, Faculty of Medicine and Health The University of Sydney Sydney New South Wales Australia; ^2^ Sydney Institute for Women, Children and their Families Sydney Local Health District Sydney New South Wales Australia; ^3^ Royal Prince Alfred Hospital Women and Babies Ambulatory Care Royal Prince Alfred Hospital Sydney New South Wales Australia; ^4^ Bioethics, Wiser Healthcare and Sydney Health Ethics, Sydney School of Public Health, Faculty of Medicine and Health The University of Sydney Sydney New South Wales Australia; ^5^ School of Women and Children's Health University of New South Wales Sydney New South Wales Australia; ^6^ Sydney School of Public Health, Faculty of Medicine and Health The University of Sydney Sydney New South Wales Australia; ^7^ Children's Hospital at Westmead Clinical School, Faculty of Medicine and Health The University of Sydney Sydney Australia; ^8^ Royal Hospital for Women Department of Maternal Fetal Medicine Sydney New South Wales Australia; ^9^ Paediatric and Perinatal Epidemiology, Children's Hospital at Westmead Clinical School, Faculty of Medicine and Health The University of Sydney Sydney New South Wales Australia; ^10^ Clinical Epidemiology, Wiser Healthcare, Sydney School of Public Health, Faculty of Medicine and Health The University of Sydney Sydney New South Wales Australia

**Keywords:** non‐invasive prenatal testing, genetic testing, prenatal diagnosis, genetic counselling, pregnancy

## Abstract

**Background:**

Non‐invasive prenatal screening (NIPS) is being increasingly used by expectant parents. Much provision of this test in Australia is occurring in clinical settings where specialised genetic counselling is unavailable, such as general practice. Potential psychosocial consequences from this kind of prenatal genetic screening remain largely unexplored.

**Aims:**

To explore clinicians' experiences with NIPS for aneuploidy, their perspectives of the benefits and harms of NIPS, clinicians' information needs, and their perceptions of the needs of expectant parents.

**Materials and Methods:**

Qualitative, semi‐structured interviews with 17 health professionals (clinical geneticists, obstetricians, genetic counsellors and general practitioners) who request and counsel for NIPS in Australian hospital and private practice settings, conducted between June 2019 and February 2020.

**Results:**

Five themes were identified relating to clinicians' perceptions and experiences of NIPS in their practice: perceived benefits of NIPS, perceived harms of NIPS (with two subthemes: clinical harms and psychosocial harms), financial and equity‐related concerns, counselling as a protective buffer against perceived harms, and clinicians' unmet education needs. While clinicians view NIPS as a useful and high‐quality screening test, especially for detection of common trisomies, many participants had concerns about how NIPS has been implemented in practice, particularly the quality (and often absence) of pre‐/post‐test counselling and the routinisation of testing for sex chromosome aneuploidies, microdeletion and microduplication syndromes.

**Conclusion:**

These findings support the need for targeted clinician training around NIPS, and for a shared decision‐making approach to support expectant parents' autonomous decisions about NIPS.

## INTRODUCTION

Non‐invasive prenatal screening (NIPS), commercially available in Australia since 2012, uses analysis of placentally derived cell‐free DNA in maternal plasma to detect genetic conditions in the fetus. NIPS was initially used to screen for trisomy 21 (T21: Down syndrome), T18 (Edwards syndrome), T13 (Patau syndrome), but is now also used to screen for a larger number of anomalies, including sex chromosome aneuploidies (SCA) and sub‐chromosomal abnormalities such as microdeletion and microduplication syndromes (MMS).^1^

Non‐invasive prenatal screening has high accuracy for the three most common trisomies, with detection (sensitivity) in singleton pregnancies of >99% of fetuses with T21 and T13, and 98% with T18. The combined false positive rate (1‐specificity) is 0.13%.^2^ Screening for SCA was added initially for monosomy X, before other SCA, including 47,XXX, 47,XXY, and 47,XYY were also added.^3^ Testing for selected MMS has been clinically available since 2013.^3^ Diagnostic accuracy for SCA and sub‐chromosomal conditions is not as high as the more common trisomies.^1^, ^2^, ^4^, ^5^ Consequently, screening for these conditions may cause a larger number of false positive results, especially if widely used in the pregnant population. Additionally, the phenotype of many of these conditions, especially the SCA, spans a wide range of neurocognitive and physical symptoms that vary in severity, with many individuals remaining undiagnosed in their lifetime.^6^ Despite recommendations from professional bodies to limit the genetic conditions routinely screened for,^7^, ^8^ the boundaries of NIPS continue to expand through new technology, including the burgeoning use of genome‐wide testing.^9^, ^10^

There are two models for implementing NIPS: a first‐tier test offered to all pregnant people, or a second‐tier test offered only if there is an increased chance result on the combined first trimester screening (cFTS) test (11–13 weeks nuchal translucency ultrasound and serum markers).^3^, ^9^, ^11^ Both models are used in Australia,^1^ with provision largely on a user‐pays basis given NIPS is not funded by Medicare. Despite limited public funding, approximately 25–30% of pregnant people in Australia currently undergo NIPS^12^ (50–75% for private obstetric care).^13^ The ad hoc, commercially driven implementation of NIPS in Australia^1^ and rapid evolution of new testing options and technologies present challenges for clinicians, introducing more complexity into clinical discussions and decision‐making around prenatal screening.^10^, ^14^ Data are lacking on Australian clinician views and experiences of NIPS, and particularly their experiences with issues that may arise when expectant parents receive a high chance or other unexpected result. To address this gap, we aimed to explore clinicians' experiences with NIPS, with a particular focus on high chance and unexpected results, benefits and harms, information needs, and clinicians' perceptions of expectant parents' needs.

## MATERIALS AND METHODS

We used a qualitative approach to capture clinicians' personal experiences and perspectives of NIPS in relation to their clinical practice. We purposively sampled clinicians who use NIPS (clinical geneticists working in public hospitals, obstetricians, (maternal‐fetal medicine (MFM) specialists) and genetic counsellors working in public or private practice, general practitioners (GPs) in shared care antenatal programs, and non‐shared care GPs), with particular focus on clinicians with greater depth of experience in dealing with high chance and unexpected NIPS results. While we originally intended to recruit more participants without specialist knowledge of NIPS, our initial interviews with clinicians in this group demonstrated limited experience with the high chance/unexpected results that were the focus of this study. Therefore, our sample was predominantly (but not entirely) made up of clinicians with high level knowledge about NIPS and genetic testing. Clinicians were recruited via email invitations, professional newsletters (Primary Health Networks and antenatal shared care programs), and author networks, from three public hospitals in New South Wales (NSW) and five private (general practice and ultrasound) practices in NSW and Queensland. Ethics approval was obtained from South Eastern Sydney Local Health District (18/283) and the University of Sydney (2019/243). Written consent was obtained pre‐interview.

The semi‐structured interview guide (Appendix S1) was developed by the multi‐disciplinary research team with expertise in MFM, clinical genetics, genetic counselling, bioethics, health policy, psychology, and clinical epidemiology, drawing on relevant literature around the potential psychosocial consequences of NIPS, and piloted with clinicians with and without specialised genetics knowledge (GPs and genetic counsellors). The interview guide was designed to be used by a non‐clinician interviewer and covered a broad range of clinical situations and experiences that may be discussed by participants, with the actual questions asked determined by the participants' previous responses. All interviews were conducted by telephone by a trained qualitative researcher (SM), audio‐recorded and transcribed verbatim. Transcriptions were cross‐checked to ensure data integrity. Data collection ceased when thematic saturation was reached (ie once newly collected data had become broadly repetitive of previously collected data in regard to the developing thematic framework).^15^

Data analysis commenced concurrently with data collection to facilitate decisions about purposive sampling and saturation. We conducted an inductive (data‐driven) thematic analysis, using the framework analysis method^16^ (Box 1). Rigour was addressed through an iterative process of constant data comparison, double coding during initial framework development, regular analysis discussions with all authors (including practising clinicians – a midwife, genetic counsellor, and MFM specialist), and cross‐checking data against thematic findings for consistency by SM and NJ.

Box 1 **Framework analysis method**

(1) Familiarisation: five authors (SM, AJN, LF, CB, KJLB) read and discussed a subset of transcripts to provide multi‐disciplinary perspectives on the preliminary coding scheme; (2) creation of an initial thematic framework; (3) indexing: transcripts were coded in Microsoft Word according to the developing thematic framework, including double coding of five transcripts by SM and a research assistant; (4) charting: themes/quotes were summarised in the framework in Microsoft Excel; and (5) mapping and interpretation: framework data were examined within and across themes and participants, summarised, and discussed with all authors.^16^

## RESULTS

Seventeen clinicians were interviewed between June 2019 and February 2020, including five obstetricians (MFM specialists), five genetic counsellors, four GPs and three clinical geneticists (Table 1). The majority of participants had a high level of knowledge of NIPS.

**Table 1:** Participant characteristics (*N* = 17)

Characteristic	Category	*n* (%)
Gender	Female	15 (88)
	Male	2 (12)
Years of practice	<10 years	10 (59)
	10–19 years	3 (18)
	>20 years	4 (24)
Clinician type	Clinical geneticist	3 (18)
	General practitioner	4 (24)
	Genetic counsellor	5 (29)
	Obstetrician (MFM)	5 (29)
Workplace	Public hospital	9 (53)
	Private practice	8 (47)
	Private ultrasound service	4 (24)
	GP practice	4 (24)

MFM, maternal‐fetal medicine specialist.

We identified five themes: perceived benefits of NIPS, perceived harms of NIPS (subthemes: clinical and psychosocial harms), financial and equity‐related concerns, counselling as a protective buffer against perceived harms, and clinicians' unmet education needs around NIPS (subtheme: relationship between education gaps and commercially driven NIPS). Table 2 presents a summary of perceived benefits and harms; Table 3 provides illustrative quotes from participant interviews.

**Table 2:** Summary of perceived benefits and harms of non‐invasive prenatal screening (NIPS)

	Perceived benefits of NIPS	Perceived harms of NIPS
Clinical	Increased accuracy for detecting T21, T18, T13 High sensitivity decreases need for invasive tests after low chance result Reduced risk of miscarriage related to potentially unnecessary invasive test following an increased chance combined first trimester screening	Changed screening behaviour (no anatomy scan conducted) False positives Low positive predictive values for some conditions Potentially unnecessary invasive tests after false positive NIPS result Potentially unnecessary termination of pregnancy after high chance NIPS result
Psychosocial	Provides reassurance Facilitates informed decision‐making Early knowledge of biological sex Facilitates bonding	False reassurance Patient anxiety and distress from poor NIPS implementation (particularly inadequate counselling) High financial cost and subsequent inequitable access to NIPS

**Table 3:** Themes and supporting quotes

Theme	Clinician quotes
Perceived benefits of NIPS	‘It's the best screening test we have ever had (…) it's really much more accurate. Much more accurate and safer (…) I think overall, it's an excellent test. I think that the benefits far outweigh the negatives.’ (ID1 clinical geneticist)
Perceived harms of NIPS *Clinical harms*	‘I tell them and I remind them again that you need the scan as well. But often I find that they are just so fixated on the blood test and they think the blood test will tell them everything.’ (ID16 GP)
	‘A number of times we find something later in the baby and they say, “But I had a NIPT†, how could you be thinking about an amnio now?” (…) trying to get that concept across that they have not just ticked off everything to do with genetics.’ (ID11 obstetrician)
	‘We've seen so many so‐called high risk 22qs (…) that were ordered by GPs, and we are yet to get a true positive. We've just done a whole bunch of diagnostic procedures for so‐called high risk normal looking babies because their GP ticked that extra box [on the NIPS request form].’ (ID6 genetic counsellor)
*Psychosocial harms*	‘A lot of the people that I see that have that increased risk results, it's related to sex chromosome aneuploidy and I think most of the people have no idea that's something that could be identified. Clearly, they are not counselled about that beforehand. I mean I've seen a few who have been really angry that – it's Iike, “Well, we did not really want this information, and now we know it, we cannot unknow it,” and they are just put in a really difficult position.’ (ID3 clinical geneticist)
Financial and equity concerns	‘Most people I see at [Hospital 1] are quite capable and happy to pay for NIPT, whereas I do not see as much of that at [Hospital 2]. There certainly are people who are comfortable and happy to pay, but certainly a lot more opting for invasive because it's covered by Medicare’ (ID4 genetic counsellor)
Counselling as a protective buffer against harms	‘She was so upset and confused. She somehow had missed that it was a Triple X, thought it was Turner's. She did not even know she'd opted into the sex chromosome; she did not even know there was a choice, but she was very upset ’cause she did not know what to do next and (…) none of this had been explained to her, none of it (…) I just felt like this patient has not been treated the way I think this should be handled. She was so lost.’ (ID6 genetic counsellor)
	‘I think in early pregnancy, there's so much information. So, this will often be given in the first or second appointment where they are getting huge amount of information. So, it can be pretty overwhelming.’ (ID19 GP)
Clinicians' unmet education needs around NIPS	‘GPs need education and clear direction and guidelines about how to just direct people about it (…) I think that all the education I've had has been about the trisomy issues, that's it, even all the latest updates, they put all the other stuff into the genetic counselling basket, which it's not. It's in our basket.’ (ID18 GP)
	‘If it's available for lots of people to recommend and request, then there needs to be clear guidance on how they should be used and training so that people are counselling appropriately, so patients know what they are or aren't getting done.’ (ID19 GP)
	‘The main impact that it's had on me is that I think there is no right answer about how it should be used in conjunction with the current tests that we already have.’ (ID7 obstetrician)
Relationship between education gaps and commercially driven NIPS	‘One of the problems is that it's very much driven by industry and by the companies. So, there's been this explosion of different tests, so obviously the technology precedes the legislation, and the implementation, and the good practice around it (…) it needs to be much better education and more timely, so that it's not 6 months or a year after all these tests have come out and the GPs are scrabbling to keep up, and try [sic] and understand what they are ordering.’ (ID3 clinical geneticist)
	‘It's kind of – seems to be pretty strongly marketed around GPs and that sort of thing, which lends itself to people doing testing possibly without good information about what test they are actually doing, which leads to all the misunderstanding and ultimately that sort of stuff can lead to patients having a bumpier ride.’ (ID15 genetic counsellor)

^†^ Non‐invasive prenatal screening (NIPS) is also commonly referred to as NIPT (non‐invasive prenatal testing).

### Perceived benefits of NIPS

Participants perceived NIPS allowed earlier, and more accurate, detection of increased chance for common autosomal aneuploidies (T21, T18 and T13), with less need for invasive tests and possible risk of miscarriage. They perceived that NIPS gave expectant parents reassurance, the ability to make informed decisions about their pregnancy, or to prepare to have a baby who may have health complications. Finding out biological sex earlier was thought to facilitate early bonding. While some participants had concerns about sex‐selective termination, none had encountered this in practice.

### Perceived harms of NIPS

Many participants perceived potential clinical and psychosocial harms from NIPS, particularly regarding screening for SCA and MMS. The GPs interviewed for this study had limited experience with high chance NIPS results and did not often identify or voice concerns about potential harms of NIPS compared to genetic counsellors, obstetricians, and clinical geneticists.

#### Clinical harms

Perceived clinical harms of NIPS included changed screening behaviour when expectant parents erroneously thought NIPS fully replaced the 12–13‐weeks (first trimester) ultrasound, potentially resulting in fetal structural abnormalities being identified at later gestation than had the first trimester ultrasound taken place. Some participants attributed this to false reassurance from a low chance NIPS result because NIPS was perceived as a diagnostic test for a broad range of conditions, rather than a screening test for specific conditions.

Participants also raised concerns about the relatively low positive predictive values for SCA and MMS. Many could recall pregnant people who experienced anxiety and had undergone potentially unnecessary invasive diagnostic tests resulting from false positive NIPS results for SCA or MMS. A couple of participants also mentioned cases where expectant parents refused a diagnostic test and chose to terminate a pregnancy based on a high chance NIPS result for SCA such as 47,XXY (Klinefelter syndrome), where false positive results on NIPS are not uncommon.

#### Psychosocial harms

Participants rarely perceived negative psychosocial consequences as inherent to NIPS itself. Rather, they attributed harms to the way clinicians offered and conducted NIPS or delivered the results (some described in Box 2). Several participants reported examples of NIPS being conducted without a viability scan, or with no/limited pre‐test or post‐test counselling. They were concerned expectant parents were not receiving information about the conditions being tested for (resulting in unanticipated outcomes), the possibility of false positive or unverified results, and whether the results would change decision‐making. Participants thought this caused distress and anxiety that could have been reduced with appropriate counselling (pre‐test and post‐test). Non‐GPs perceived that insufficient counselling was more common when NIPS had been requested through clinicians without specialist genetic knowledge, such as GPs and general obstetricians.

Box 2 **Case studies of perceived psychosocial harms related to NIPS.**

The following case studies are based on direct quotes from participants, and illustrate perceived psychosocial harms related to how NIPS may be offered, conducted and the delivery of results. They are not intended to depict typical experiences of NIPS.

**Case 1 Marina and Paul**

Marina and her husband Paul presented to hospital without a referral requesting a termination of pregnancy after Marina's NIPS test, which had been requested by her GP, returned an increased chance result for a rare SCA. Their GP had advised them that the NIPS result was >99% accurate. Hospital staff directed the couple to a genetic counsellor. The counsellor spent time with Marina and Paul attempting to ‘undo’ the understanding that they had taken from the GP, explaining to them that NIPS is not highly accurate for the condition they received an increased chance result for, and strongly recommending that they return for an invasive test to confirm the diagnosis. At the end of the counselling session, Marina was booked in for an invasive diagnostic test, to take place in 1–2 weeks. Marina did not return for the diagnostic test, and when the counsellor contacted the couple to follow‐up, she was informed that they had arranged a private termination.^1^

**Case 2: Maya**

Maya contacted a private ultrasound practice to request a second NIPS test after receiving an increased chance result for SCA on a NIPS test requested by her GP. Maya had not been referred to the service by her GP, she contacted the practice independently. When the genetic counsellor called Maya to explain that the service does not repeat NIPS testing as a rule, Maya was upset and confused. In speaking to Maya about her NIPS results, the counsellor realised that Maya had actually received an increased chance result for a different condition from what she had initially described; she had not understood the results as delivered to her by her GP. Maya told the counsellor she had not known that she was being tested for SCA, and she had not realised she had a choice about what to test for. She told the counsellor that she did not know what steps to take after receiving an increased risk result. The genetic counsellor felt that Maya's situation had been poorly handled and arranged for her to undergo diagnostic testing at a public hospital.^1^

### Financial and equity concerns

Participants raised concerns that the lack of public funding for NIPS means uptake is higher among those with higher incomes, potentially contributing to inequities and discrepancies in care provision. They reported that people with intermediate‐chance results from cFTS who could not afford NIPS as a second‐tier screening test instead opted for publicly funded invasive diagnostic procedures, with greater risk of miscarriage. Some participants had concerns that inadequate pre‐test counselling meant expectant parents who did choose NIPS were vulnerable to unnecessary financial loss if they chose to purchase add‐ons (eg MMS) without knowledge of their limitations. Some speculated that Medicare‐funded NIPS may be particularly beneficial for those in regional and rural areas where there is limited access to invasive testing with chorionic villus sampling or amniocentesis. Most thought that NIPS should be (at least partly) funded by Medicare.

### Counselling as a protective buffer against harms

Most participants believed that while some anxiety and distress around NIPS results were inevitable, pre‐/post‐test counselling that clearly explains NIPS as a screening test and describes alternate screening options, the conditions being tested for, the options for add‐on tests, and the limitations of NIPS (particularly for SCA and MMS), could protect against many of the perceived negative consequences of NIPS. A lack of appropriate pre‐test counselling, often combined with poor communication of high chance NIPS results, was a common factor in most cases where participants perceived psychosocial harms had occurred.

At the same time, many participants, particularly GPs, reported limited time for pre‐test counselling in the context of first trimester antenatal appointments. Participants perceived that information provided during pre‐test counselling was often not retained by those who expected low chance results.

### Clinicians' unmet education needs around NIPS

Many participants identified unmet education and information needs for clinicians (particularly those without genetics training) around NIPS. Some participating GPs suggested there should be mandatory training for GPs on how to discuss NIPS and disseminate the results. When directly asked, most participants responded that a decision aid to support shared decision‐making could be a valuable tool for some clinicians, particularly in clinical settings where extensive genetic counselling was not available. Participants highlighted the need for guidelines on providing pre‐ and post‐test counselling, particularly on SCA.

### Relationship between education gaps and commercially driven NIPS

Some participants drew a connection between the need for more consistent information/education for clinicians and the commercially driven nature of NIPS in Australia. They perceived the lack of regulatory oversight and the differences in NIPS technologies and screening options offered by different commercial providers as driving variation in practice and implementation of expanded screening technologies not recommended by professional guidelines. Some were concerned that the commercialised nature of NIPS has resulted in testing companies directing marketing to GPs to encourage offering of NIPS, without sufficient investment in the education required to ensure appropriate information and counselling is offered alongside the test.

## DISCUSSION

Clinicians in this study viewed NIPS as a clinically useful screening test, especially for common trisomies. However, many had concerns about how NIPS is being implemented in practice, including the quality of counselling, lack of clinician training around NIPS, and the commercially led implementation of NIPS in Australia. There were also concerns about routinely testing for SCA and MMS (often offered on an opt‐out basis). Both GPs and non‐GPs voiced the need for increased training about NIPS to be made available to clinicians without specialist genetics knowledge. These findings are largely in line with research into clinicians' views of NIPS in other countries,^14^, ^17^, ^18^ regardless of whether NIPS is offered as a first‐ or second‐tier test. Models of implementation vary globally, with second‐tier testing for trisomies 21, 18 and 13 only tending to be more common in countries where NIPS is publicly funded.^19^ In contrast, the ad hoc implementation of NIPS in Australia,^1^ outside of any formal screening or public funding framework, has led to the choice of technologies available to clinicians and expectant parents being largely driven by industry rather than regulators, consistent with how NIPS has been implemented in countries with market‐based healthcare systems such as the USA.^19^

Our findings suggest that clinicians who see expectant parents after they have received a high chance result perceive that many expectant parents are not receiving adequate counselling from the clinician who requests the test (often a non‐specialist), and may not sufficiently understand the implications of NIPS. This is supported by previous Australian research that found that a significant minority of NIPS users gave a neutral or negative response when asked about the adequacy of the pre/post‐test counselling they received.^20^ We have also conducted research with people who have undergone NIPS to confirm how they understand NIPS and its implications; these results will be separately reported. The majority (75%) of pregnant people in Australia receive public hospital‐based maternity care^21^ but for many, early antenatal care, including offering of NIPS, is often provided by GPs. This ‘mainstreaming’ of NIPS into general practice needs to be accompanied by increased training and support for GPs who are otherwise unlikely to have the specialist genetic knowledge required to provide appropriate counselling. They, and other clinicians, may also be better able to deliver high‐quality counselling if this was supported through a funding item on the Medicare Benefits Schedule (MBS), to allow the necessary time spent within consultations.

We found support for a shared decision‐making approach to NIPS, which may not only facilitate high‐quality informed consent for NIPS,^6^, ^10^ but also help expectant parents make decisions aligning with their values and their own specific context. To achieve this, pre‐test counselling should include values clarification as well as provide information about NIPS. Most participants in this study focused on the attributes of the test when discussing what they considered to be optimal pre‐test counselling, rather than exploring the reasons a person might choose to have NIPS, and what they might hope to gain from the results. A shift in focus of counselling for NIPS is needed, to move away from a granular focus on the technical aspects of the test, to instead emphasise what options NIPS might give rise to, and how expectant parents feel about them. Some prenatal screening decision aids are already available,^22^, ^23^ but their main focus is screening for T21. Developing or adapting/updating existing decision support tools for NIPS that incorporate extended test panels is a key area where further research is needed. There is also a need for developing and delivering educational packages for clinicians who order and counsel for NIPS.

However, shared decision‐making should not be used to compensate for the inherent limitations of NIPS to screen for SCA and MMS. This may inappropriately transfer responsibility for deciding about a test to parents. There is a duty to protect parents from the current risks inherent in NIPS. As such, additional policy options should be considered, including making the offer of screening for SCA and MMS from NIPS strictly opt‐in (with specific counselling) and/or identifying regulatory mechanisms to ensure that current professional guideline recommendations against routinely using NIPS to detect MMS and genome‐wide chromosome abnormalities^7^ are followed in practice. There also needs to be more specific guidance for GPs on the use of NIPS for screening (those currently available are focused on screening for T21^24^, ^25^), and a national system to collect routine data on NIPS requests. This will allow ongoing evaluation of NIPS uptake, and, through data linkage to other administrative datasets, a better understanding of potential downstream consequences (both benefits and harms). Finally, should NIPS become listed on the MBS, a practice note for the item could signal best practice: that the test should be offered by practitioners with appropriate training or expertise and done in conjunction with high‐quality counselling.

Strengths of this study include a rigorous analysis process, and the involvement of a multi‐disciplinary team throughout the research process. Limitations include that self‐report may differ from actual practice, and all participants worked in metropolitan areas with predominantly middle‐ to high‐income populations. Our sample did not include midwives (although our research team did) or general obstetricians, and the interview guide was developed without the input of clinicians from these two groups. Our findings reflect the fact that our sample was predominantly made up of clinicians with a high level of knowledge about NIPS and genetic testing, who regularly care for expectant parents who have received high chance NIPS results. Their experiences, views and perceptions of psychosocial harm are likely to be distinct from clinicians without specialised knowledge of genetics whose experience with NIPS may be more centred on pre‐test counselling and whose patients generally receive low chance NIPS results. While our sample included GPs without specialist knowledge, it is unlikely that we reached thematic saturation in regard to the experiences and needs of GPs around NIPS. Further research is needed to ascertain the knowledge, views, and specific needs of clinicians without specialised genetics knowledge (including GPs, midwives and general obstetricians), if new educational resources are to be developed for them.

Our findings support the need for targeted training around NIPS, a shared decision‐making approach to support expectant parents' autonomous decisions, and policy initiatives to protect against potential harms while extending potential benefits.

## Funding

This work was supported by funding from the Australian National Health and Medical Research Council (NHMRC) through Centre for Research Excellence (#1104136) and Investigator (#1174523) grants.

## Supporting information


**Appendix S1** Interview topic guide.

## Endnote

^1^
All names are pseudonyms. Some details have been changed for anonymity purposes.
