# Supplementary material for: Clinician views and experiences of non‐invasive prenatal genetic screening tests in Australia
Source: Aust N Z J Obstet Gynaecol. 2022 May 10;62(6):830–7. doi: 10.1111/ajo.13533 (PMC10946867; doi:10.1111/ajo.13533)
Supplement: Supplementary file 1 — Appendix S1 Interview topic guide. [file AJO-62-830-s001.docx]

**Semi-structured Interview Guide – Clinicians**

The interview will be conducted as a conversation facilitated by the interviewer and focused around the following broad areas. Examples of specific questions are given but actual questions asked, and their order, will depend on the answers given by individual participants. The interviewers are trained to respond to cues and to probe appropriately.

1. **General information about health professional**

Example question:

- 1. Tell me about yourself and your work

1. **Experiences and opinions/perceptions about NIPS**

Example questions:

- 1. Can you tell me about your experience with Non-Invasive Prenatal Screening (NIPS) in your practice?
  2. What is your personal opinion about NIPS?
  3. What do you think are the main advantages of NIPS?
  4. What do you think are the disadvantages of NIPS?
  5. How do you talk about the results of NIPS with your patients?
  6. What kind of impact does NIPS have on your patients?
  7. Do these tests impact you/your practice?
  8. Tell me about a time that a patient of yours has experienced a false positive result / inconclusive result?

1. **Use of NIPS generally**

Example questions:

- 1. How do you think NIPS is best used among pregnant women in general?
  2. Do you think this test should be offered to all pregnant women as a first line screening test? Why/why not?

1. **Concluding questions**

Example questions:

- 1. Based on your experience and the discussion we’ve had today, how do you think these tests should be offered / conducted? Why?
  2. With new genetic tests on the horizon, what do you think we can learn from how previous tests, such as those we discussed today, have been implemented?
  3. If a Decision Aid was being developed around the decision to have NIPS, would you find this useful? What information do you think would be useful for you, and for your patients?
  4. Is there anything else you’d like to add before we finish?
